# Multiplex CRISPR Mutagenesis of the Serine/Arginine-Rich (SR) Gene Family in Rice

**DOI:** 10.3390/genes10080596

**Published:** 2019-08-07

**Authors:** Haroon Butt, Agnieszka Piatek, Lixin Li, Anireddy S. N. Reddy, Magdy M. Mahfouz

**Affiliations:** 1 Laboratory for Genome Engineering and Synthetic Biology, Division of Biological Sciences, King Abdullah University of Science and Technology, Thuwal 23955-6900, Saudi Arabia; 2 Program in Molecular Plant Biology, Program in Cell and Molecular Biology, Department of Biology, Colorado State University, Fort Collins, CO 80523-1062, USA

**Keywords:** splicing, alternative splicing, SR proteins, genome engineering, multiplex targeting, CRISPR/Cas9

## Abstract

Plant growth responds to various environmental and developmental cues via signaling cascades that influence gene expression at the level of transcription and pre-mRNA splicing. Alternative splicing of pre-mRNA increases the coding potential of the genome from multiexon genes and regulates gene expression through multiple mechanisms. Serine/arginine-rich (SR) proteins, a conserved family of splicing factors, are the key players of alternative splicing and regulate pre-mRNA splicing under stress conditions. The rice (*Oryza sativa*) genome encodes 22 SR proteins categorized into six subfamilies. Three of the subfamilies are plant-specific with no mammalian orthologues, and the functions of these SR proteins are not well known. The clustered regularly interspaced short palindromic repeats (CRISPR)/CRISPR-associated protein 9 (Cas9) system is a genome engineering tool that cleaves the target DNA at specific locations directed by a guide RNA (gRNA). Recent advances in CRISPR/Cas9-mediated plant genome engineering make it possible to generate single and multiple functional knockout mutants in diverse plant species. In this study, we targeted each rice SR locus and produced single knockouts. To overcome the functional redundancy within each subfamily of SR genes, we utilized a polycistronic tRNA-gRNA multiplex targeting system and targeted all loci of each subfamily. Sanger sequencing results indicated that most of the targeted loci had knockout mutations. This study provides useful resource materials for understanding the molecular role of SR proteins in plant development and biotic and abiotic stress responses.

## 1. Introduction

Plants are constantly exposed to diverse and changing environmental conditions. Changes in their habitat can adversely affect their growth and development [1,2]. Therefore, plants have evolved numerous molecular mechanisms to survive under environmental fluctuations. Among these, regulation of gene expression is a major form of adaptation in response to biotic and abiotic stresses [3,4]. Pre-mRNA splicing is primarily a co-transcriptional process conserved among eukaryotes. It is a key step in gene regulation and is performed by the spliceosome, a large macromolecular complex. Spliceosomes comprise of five small nuclear ribonucleoproteins (snRNPs: U1, U2, U4/U6, and U5) and are assisted by ~200 accessory associated proteins [5,6]. Spliceosome assembly occurs independently on each intron, beginning with the binding of the U1 snRNP and U2 auxiliary factor (U2AF) to the 5′ and 3′ splice sites, respectively. The resulting scaffold, termed the E complex, recruits the U2 snRNP to the branch-point sequence, forming pre-spliceosome complex A. Subsequently, the U5-U4/U6 tri-snRNP is recruited to the assembling spliceosome to form the B complex. Spliceosome assembly reaches completion upon subsequent conformational rearrangements that yield the catalytically active spliceosomal C complex, which catalyzes the transesterification reactions that lead to the excision of the intervening intron and ligation of adjoining exons [7,8].

One of the important aspects of alternative splicing (AS) of pre-mRNA is that it enhances the coding capacity of the genome from multiexon genes. Regions encoding different protein domains can be selectively retained or excised from the mature mRNA so that a single gene can generate multiple distinct proteins that may vary greatly in structure, function, and other properties [9,10]. AS occurs when splice sites are differentially selected and multiple isoforms are produced from the same transcript. AS is fine-tuned by a group of RNA-binding proteins that bind the sequence signals in the pre-mRNA and guide the spliceosome complex for splicing regulation. Serine/arginine-rich proteins are important RNA-binding proteins that function as splicing factors, determine splice sites (SSs), and regulate pre-mRNA splicing under normal and stress conditions [11,12]. Various environmental stresses influence widespread changes in the AS of pre-mRNAs, especially from stress-responsive genes [13,14,15,16]. Members of the SR protein family, such as SRSF1 and SRSF2 in animals, were examined for their crucial functions in constitutive splicing as they were discovered to recruit U1 snRNP to the 5′ SS and U2 snRNP binding to the 3′ SS. Moreover, they bridge the interaction between these initial SS recognition occurrences in the pre-spliceosome and the mature spliceosome [17,18]. Several studies have corroborated the role of SR proteins in stress responses. The loss-of-function mutants of *rs40* and *rs41*, *scl30a*, and *sr45* showed hypersensitivity to salt and abscisic acid (ABA) stress in *Arabidopsis* [19,20,21]. Moreover, the *sr34b* mutant showed increased sensitivity to cadmium (Cd) stress and expression of *SR34b* increased under Cd stress [22]. The SR proteins also play important roles in mineral nutrient homeostasis in rice (*O. sativa*) [23]. The biological roles of SR proteins in animals are further extended to mRNA nuclear export, mRNA stability, genome maintenance, and translation [24,25,26].

The clustered regularly interspaced short palindromic repeat (CRISPR)/CRISPR-associated protein (Cas) adaptive immunity system has been harnessed for genome editing applications across eukaryotic species [27,28,29]. For targeted genome engineering, CRISPR/Cas9 is modified into two components: A single-guide RNA (sgRNA) and a Cas9 endonuclease, which is guided by the sgRNA to a specified genomic locus. The Cas9 endonuclease cleaves a specific genomic DNA sequence by introducing double-strand breaks (DSBs) at the target site. These DSBs can be repaired by one of two endogenous mechanisms: Nonhomologous end joining (NHEJ) or homology-directed repair (HDR). The repair of DSBs via NHEJ often leads to the formation of small insertion/deletion (InDel) mutations. These InDel mutations can disrupt coding or regulatory sequences of the target gene, resulting in loss-of-function mutations. Repair of double-strand breaks by HDR requires the simultaneous delivery of a DNA repair template that carries the desired modification to be incorporated into the repaired locus. This powerful molecular tool is used for basic research [30,31], medical applications [31,32,33], and crop improvement [34,35].

In this study, we utilized the CRISPR/Cas9 technology to target all members of the *SR* gene family in rice. We produced single knockout mutants and multigene mutants to overcome the functional redundancy within each subgroup. Sanger sequencing analysis of the T_0_ generation indicated that most of the single targeted genes had knockout mutations.

## 2. Material and Methods

### 2.1. Plant Materials and Vector Construction

*O. sativa* L. ssp. *japonica* cv. Nipponbare was used for all experiments. The expression of Cas9 was driven by *OsUBIQUITIN*. The pRGEB32 vector was used for callus transformations. The sgRNAs were designed to target the 1st or 2nd exon of each locus. The sgRNA was synthesized as a polycistronic tRNA-gRNA (PTG) fragment. For multiplex targeting, synthetic PTG fragments contained more than one sgRNA that were separated by tRNA. Each fragment had BsaI overhangs for cloning under the *OsU3* promoter in pRGEB32 [36]. The sequences of the PTG fragments are given in Table S1.

### 2.2. Agrobacterium tumefaciens-Mediated Transformation of Rice

*Agrobacterium tumefaciens*-mediated rice transformation was performed using the strain EHA105 as described previously [30,37]. Briefly, callus induction is done at 2N6 media. The *Agrobacterium* cells set to a density of OD600 = 0.3 incubated with calli for 5 min. After co-cultivation under dark at 25 °C for three days, rice calli were selected on media supplemented with Hygromycin (50 mg/L) and Timentin (200 mg/L). After the screening stage, actively growing calli were sub-cultured onto a regenerative medium for regeneration under continuous light. After 2–3 weeks, transgenic seedlings were transferred to sterile plastic containers containing fresh rooting medium and grown for 2–3 weeks before being transferred into soil. Transgenic rice plants were grown in a growth chamber at 28 °C with a 16 h light/8 h dark cycle.

### 2.3. Genotyping of the SR Mutant Plants

After one week, when plants were established on soil, DNA was extracted from leaf samples frozen in liquid nitrogen after collection. Frozen plant material was homogenized to a fine powder. Afterwards, 300 µL of the extraction buffer (200 mM Tris-Cl (pH 7.5), 250 mM NaCl, 25 mM EDTA (Ethylenediaminetetraacetic acid) and 0.5% SDS (Sodium dodecyl sulfate)) were added to the sample, vortexed to mix the sample with the buffer. 300 µL of PCI (Phenol:Chloroform:IsoAmylalcohol 25:24:1) were added, vigorously vortexed and spun down for 10 min at 13,000 rpm and room temperature (RT). The aqueous phase (upper) was precipitated with isopropanol (80% of total volume) and 3M sodium acetate (10% of total volume) incubated for 10 min at RT and then centrifuged at 13,000 rpm for 10 min at RT. The supernatant was removed and the pellet was washed with 500 µL of 70% ethanol and successive centrifugation for 5 min at 13,000 rpm. The ethanol was completely removed and the pellet was air-dried for 15–20 min. The pellet was dissolved in 50 µL TE (Tris-EDTA) buffer. 1–2 µL were used for further PCR. PCR was conducted using gene-specific primers. Purified PCR products were cloned using the CloneJET PCR Cloning Kit (K1231, Thermo Fisher Scientific, Waltham, MA, USA). The ligation mixture was directly used for transformation into *Escherichia coli* competent cells. Recombinant clones were selected and DNA was purified and subjected to Sanger sequencing. The oligo sequences used for genotyping are listed in Table S2.

## 3. Results

### 3.1. Targeted Multiplex Genome Engineering of the SR Gene Family in Rice

**Figure 1:**
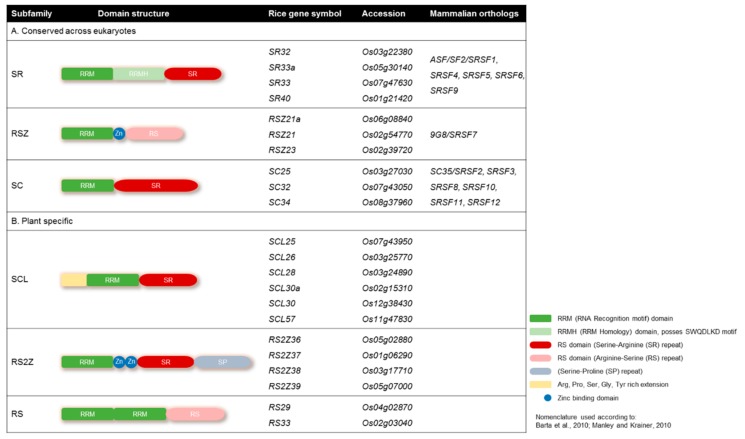
Domain structures of the serine/arginine-rich (SR) proteins. Domain architecture of the SR protein subfamilies with their mammalian orthologs. (**A**) Highly conserved eukaryotic SR protein subfamilies; (**B**) plant-specific SR protein subfamilies. Rice has 22 SR proteins, 10 of which are conserved among eukaryotes, while the other 12 are plant-specific. The nomenclature adopted from [38,39].

Plant SR proteins are defined as having one or two N-terminal RNA binding domains (RBDs), also called RNA recognition motifs (RRMs), and a C-terminal arginine/serine-rich (RS) domain with SR or RS dipeptide content [38,39] (Figure 1). The N-terminal domains are responsible for binding with the mRNA transcript, and the C-terminal region is responsible for protein-protein interaction during the splicing process. Rice has 22 members of SR proteins, the most among all known species, which can be categorized into six subfamilies. Three of these subfamilies (SR, RSZ, and SC) are conserved among eukaryotes, while the rest (SCL, RS2Z, and RS) are plant-specific (Figure 1). The plant-specific subfamilies have unique structural features; for example, the SCL subfamily is similar to the SC subfamily but possesses a unique N-terminal domain rich in charged amino acids (Figure 1). The RS2Z subfamily has two zinc knuckles instead of one in RSZ members. RS2Z members also have an additional serine- and proline-rich C-terminal domain. The second RRM domain of RS subfamily members is deficient in the SWQDLKD motif and possess RS dipeptides in the C-terminus (Figure 1).

**Figure 2:**
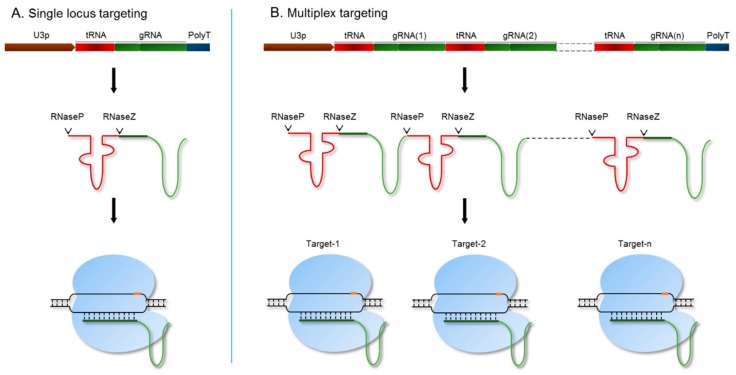
The polycistronic tRNA-gRNA (PTG)/ CRISPR/CRISPR-associated protein 9 (Cas9) system for targeting single or multiple genes. The synthetic PTG molecule consists of a tRNA-gRNA unit. (**A**) For targeting a single locus, PTG was expressed under the OsU3 promoter. The PTG fragment is spliced by RNaseP and RNaseZ, and the sgRNA is released to further guide the Cas9 endonuclease to its genomic target; (**B**) multiple loci were targeted using the PTG system. Mature sgRNAs are released by the cleavage activity of RNaseP and RNaseZ. Each sgRNA directs the Cas9 endonuclease to its target genomic region.

For targeted engineering of the SR genomic regions, we utilized a polycistronic tRNA-gRNA (PTG)/Cas9 system, which allows for precise processing of functional gRNA [36] (Figure 2). This system is expressed under the rice *OsU3* promoter with a poly(T) terminator. The endogenous machinery of RNaseP and RNaseZ cleaves the PTG transcript by recognizing transfer RNA (tRNA) sequences [36]. As a result, the gRNA is released as a single molecule and loaded onto the Cas9 endonuclease to target a specific genomic region. To target a single locus, we used a synthetic fragment of the tRNA-gRNA-terminator with BsaI overhangs to clone under the *OsU3* promoter (Table S1). We designed a PTG fragment for each SR member and cloned each into the binary vector pRGEB32 [36]. We then performed *A. tumefaciens*-mediated rice callus transformation for each of the individual targets to produce single-gene knockout mutants. Genes within each subfamily of the SR family have high sequence similarity and probably have redundant functions. Therefore, we designed and synthesized a single PTG fragment with gRNAs to target all the members of a subfamily (Figure 2B). Each gRNA targeting a single locus is released after processing from the PTG transcript and forms a complex with the Cas9 endonuclease to simultaneously target all the loci of an *SR* subfamily [36] (Figure 2).

The gRNAs were specifically designed in the first or second exon to perturb the function of the protein. After the rice callus transformation, the plants were transferred to soil. Once plants were established on soil for 10 days the DNA was extracted from the leaves and genotyped by Sanger sequencing.

### 3.2. The SR Subfamily

**Figure 3:**
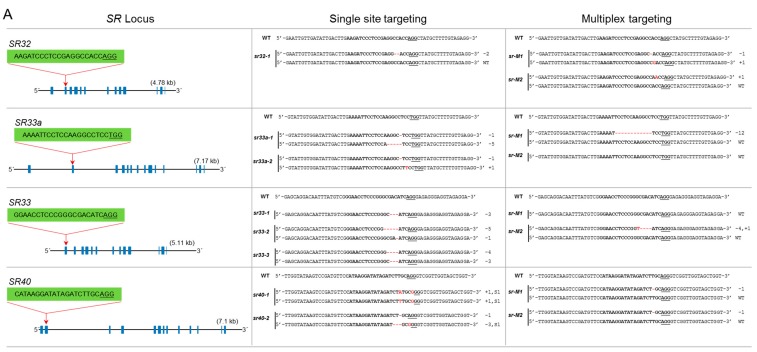

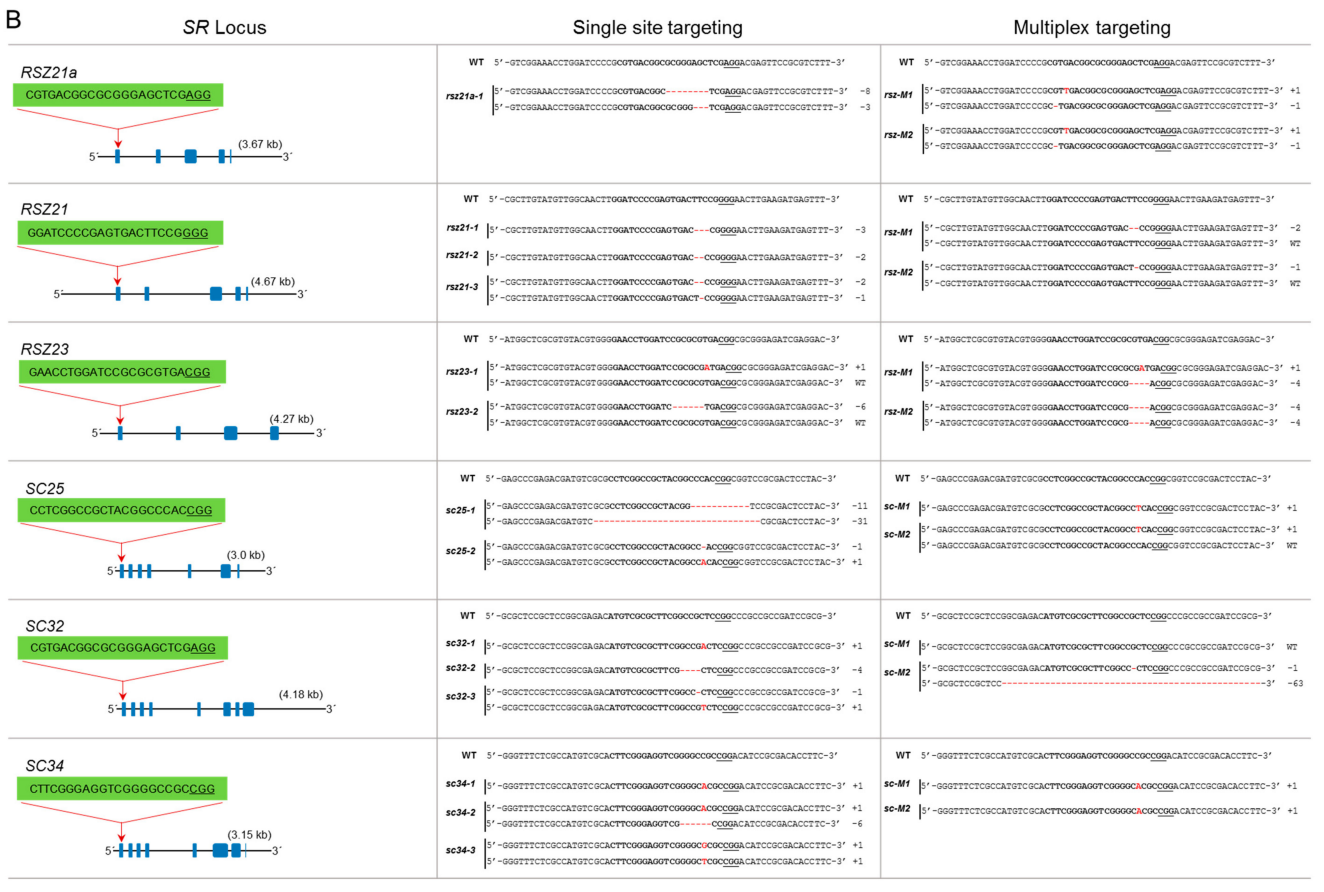

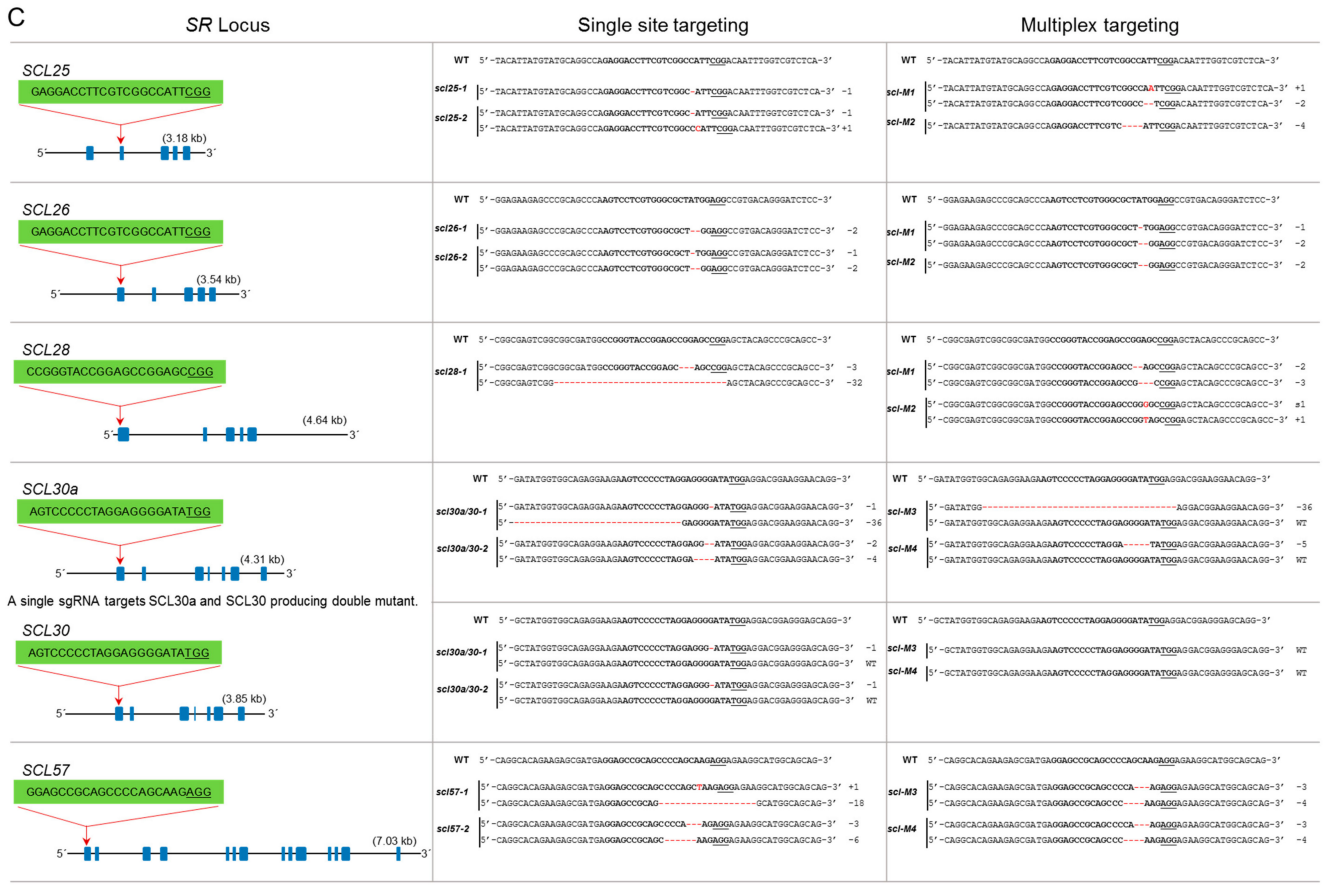

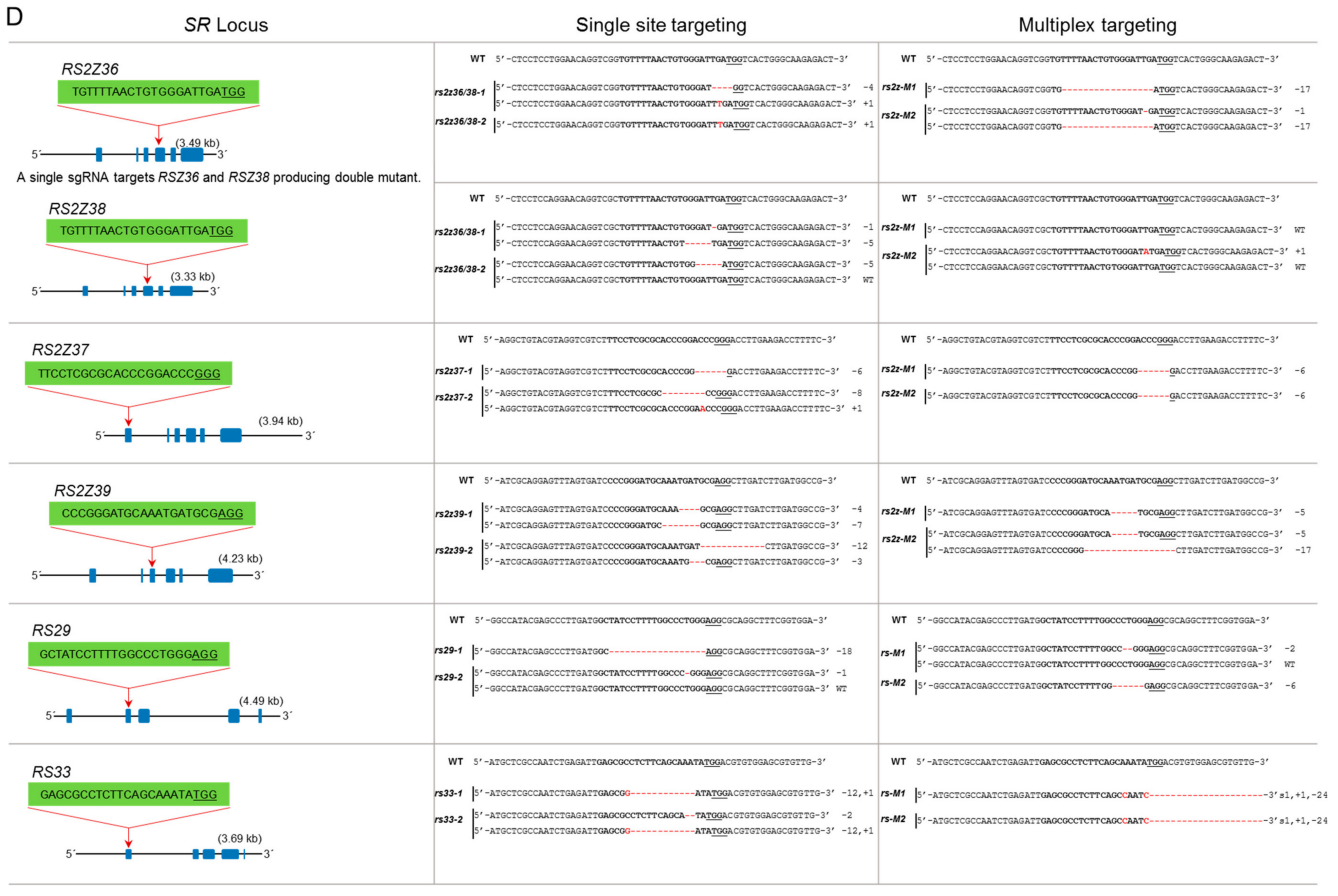
Single and multiplex mutagenesis of SR proteins using the PTG system. sgRNAs were designed to target each *SR* locus mostly in the first or second exons. The sgRNA target sequence is indicated in bold. The protospacer adjacent motif (PAM) sequence is underlined. Blue boxes indicate exons, and lines represent introns. Multiplex targeting was done for each subgroup of the SR family, in which all loci were targeted simultaneously. The multiplex mutants were named according to their subfamily name preceded by “*M*” for “multiplex.” (**A**) Single and multiplex mutants are identified for the SR subfamily; (**B**) single and multiplex mutants for RSZ and SC subfamilies; (**C**) the SCL subfamily has six members. Multiplex targeting was done for *SCL25/26/28* and *SCL30a/30/57. SCL30a* and *SCL30* are highly conserved; a single sgRNA was used to target both loci; (**D**) mutagenesis of RS2Z and RS subfamilies. *RS2Z36* and *RS2Z38* are highly conserved, and a single sgRNA was used to target both loci.

The SR subfamily has four members: *SR32*, *SR33a*, *SR33*, and *SR40*. These proteins have the SWQDLKD motif and have the mammalian orthologs ASF/SF2/SRSF1. *SR32* has 13 exons, and we targeted the 2nd exon. We genotyped 10 lines but did not recover a mutant for this locus (Figure 3A). *SR33a* has 14 exons. The gRNA was designed to target the 2nd exon, and we successfully recovered knockout biallelic mutants for this gene. *SR33* has a high sequence similarity to *SR33a* and contains 13 exons. We used a gRNA targeting the 1st exon and produced three mutants. *sr33-1* is an in-frame mutant and *sr33-3* is a biallelic mutant with in-frame and knockout mutations. *sr33-2* is a complete knockout mutant (Figure 3A). *SR-40* has 13 exons, and we targeted the 2nd exon; only the *sr40-1* mutant is a complete knockout mutant. In the multiplex targeting experiment, we did not recover a complete knockout mutant for all four loci. The *sr-M1* mutant has a complete knockout mutation for *SR32* but was heterozygous for a mutated and wild-type allele for *SR33a* and *SR40*, while no mutation was generated for *SR33*. The *sr-M2* mutant is heterozygous for a mutated and wild-type allele for *SR32*, *SR33*, and *SR-40* but has no mutation for *SR33a* (Figure 3A).

### 3.3. The RSZ Subfamily

The RSZ subfamily has three members: *RSZ21a*, *RSZ21*, and *RSZ23*. This subfamily is characterized by one zinc knuckle and has an *SRSF7/9G8* motif found in mammalian counterparts. *RSZ21a* has five exons, and we targeted the 1st exon. The mutant line is biallelic with one allele containing an in-frame mutation. *RSZ21* was targeted at its 1st exon, and two monoallelic and one biallelic mutants were generated. *rsz21-1* is a monoallelic in-frame mutant, while the other two mutants are complete knockouts. *RSZ23* contains only four exons, and the gRNA targeted the 1st exon. We did not recover a single knockout mutant for this gene; both mutants for this locus are heterozygous and have a wild-type allele (Figure 3B). In multiplex targeting, we did not recover a complete triple knockout mutant. The *rsz-M1* and *rsz-M2* mutants are biallelic mutants for *RSZ21a* and *RSZ23* but heterozygous for *RSZ21* containing a mutated allele and the wild-type allele (Figure 3B).

### 3.4. The SC Subfamily

The SC subfamily has three members: *SC25*, *SC32*, and *SC34*. This group has one RRM domain and an SR domain. The mammalian orthologs are SRSF2/SC35. *SC25* has seven exons, and the gRNA was designed to target the 1st exon. We generated two single knockout biallelic mutants for this locus. *SC32* has eight exons, and we targeted the 1st exon. The mutants for this locus are all complete knockouts and one is a biallelic mutant. *SC34* contains eight exons, and the gRNA was designed to target the 1st exon. The single mutants for this locus are complete knockouts except for the *sc34-2* mutant, which has one allele with an in-frame deletion (Figure 3B). During multiplex targeting, we produced the *sc-M1* mutant, which is a complete knockout for *SC25* and *SC34* but has no mutation for *SC32*. The *sc-M2* mutant has a knockout mutation for *SC34* and a biallelic mutation for *SC32* with one in-frame deletion of 21 amino acids and is heterozygous for *SC25* containing a mutated allele and the wild-type allele (Figure 3B).

### 3.5. The SCL Subfamily

This is the largest subfamily of *SR* genes with six members: *SCL25*, *SCL26*, *SCL28*, *SCL30a*, *SCL30*, and *SCL57*. This is a plant-specific subfamily but is similar to *SC35*; hence, it is named SC35-lik3 (SCL). This group has an N-terminal extension of charged amino acids. *SCL25* has five exons, and we targeted the 2nd exon. We generated single knockout mutants for this locus. Similarly, *SCL26* has five exons, and the gRNA targeted the 1st exon. The mutants for this locus have one- and two-nucleotide deletions. *SCL28* has five exons, and the gRNA target site was in the 1st exon. The only recovered mutant is biallelic and has a deletion of 32 nucleotides and an in-frame deletion of three nucleotides (Figure 3C) *SCL30a* and *SCL30* have highly similar nucleotide sequences; a single gRNA was designed to target both loci at their 1st exons. The *scl30a/30-1* mutant is biallelic for a mutation in *SCL30a* but heterozygous for *SCL30* with a mutated and a wild-type allele at this locus. Similarly, *scl30a/30-2* is a knockout for *SCL30a* but is heterozygous for *SCL30* (Figure 3C). *SCL57* contains 14 exons, and the gRNA targeted the 1st exon. The mutants produced are biallelic with different in-frame deletions (Figure 3C). For efficient multiplex mutagenesis, we targeted only three loci of this subfamily for each multiplex reaction. *scl-M1* is a triple mutant for *SCL25/26/28* and has only one in-frame deletion for *SCL28*. *scl-M2* is a triple knockout mutant for all three loci, *SCL25/26/28* (Figure 3C). The *scl-M3* mutant is heterozygous with an in-frame mutation for *SCL30a* and a wild-type allele, a biallelic mutation for *SCL57*, and no mutation for *SCL30*. The *scl-M4* mutant is heterozygous for *SCL30a*, has no mutation for *SCL30*, and a biallelic mutation for *SCL57* (Figure 3C).

### 3.6. The RS2Z Subfamily

Proteins of this plant-specific family have two zinc knuckles and a characteristic serine/proline-rich region at their C termini. This subfamily has four members: *RS2Z36*, *RS2Z37*, *RS2Z38*, and *RS2Z39*. *RS2Z36* and *RS2Z38* have high sequence similarity, and a single gRNA was designed to target both loci at their 4th exons. *rs2z36/38-1* is a double mutant for *RS2Z36* and *RS2Z38*, while *rs2z36/38-2* is a knockout mutant for *RS2Z36* but was heterozygous for *RS2Z38* with a mutated allele and a wild-type allele. *RS2Z37* has six exons, and the gRNA targeted the 1st exon. *rs2z37-1* is a monoallelic in-frame mutant, but *rs2z37-2* is a complete knockout biallelic mutant. *RS2Z39* has six exons, and the gRNA targeted the 3rd exon. The mutants recovered for this locus are homozygous, but only the *rs2z39-2* mutant has an in-frame mutant allele (Figure 3D). During multiplex targeting, we recovered triple mutants for *RS2Z36*, *RS2Z37*, and *RS2Z39*, but the *RS2Z38* locus was not mutated (*rs2z-M1*) and the other was heterozygous, containing a wild-type allele and a mutated allele (*rs2z-M2*) (Figure 3D).

### 3.7. The RS Subfamily

The RS subfamily is plant-specific and has only two members in rice. *RS29* has five exons, and the gRNA targeted the 2nd exon. We recovered two mutants, a monoallelic mutant with an in-frame deletion of 18 nucleotides and a heterozygous mutant with a mutated allele and the wild-type allele. *RS33* has five exons, and the target site for the gRNA was in the 1st exon. The mutants for this locus are complete knockouts (Figure 3D). We did not recover a double knockout mutant for these loci during multiplex targeting. The *rs-M1* mutant is heterozygous for *RS29* and has an in-frame deletion for *RS33*. Similarly, the *rs-M2* mutant has in-frame deletions for *RS29* as well as for *RS33* (Figure 3D).

### 3.8. Heritability in the Seed Progeny of SR Family Mutants

**Figure 4:**
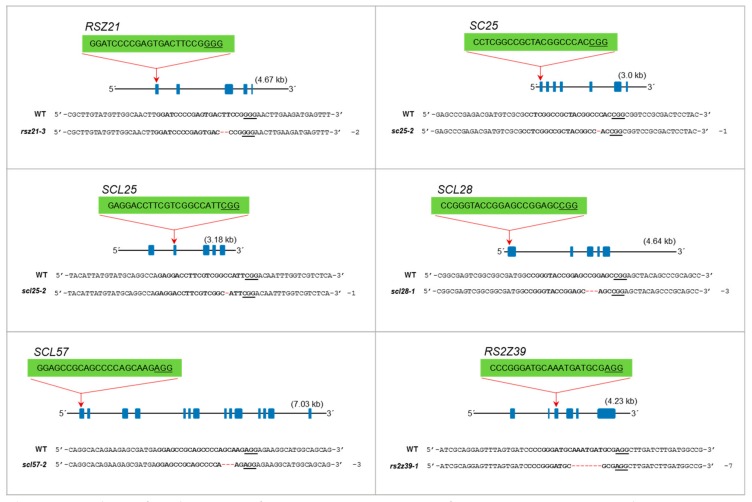
Analysis of seed progeny of SR mutants. Genotyping of SR gene mutations in seed progeny. The heterozygous parent plants were segregating to homozygous monoallelic mutants for *RSZ21, SC25, SCL25, SCL28, SCL57* and *RS2Z39*.

Recovery of homozygous mutants in the progeny is essential to use these mutants for phenotypic analysis to understand the responses of these mutant to growth, development, abiotic and biotic stress cues. To confirm the heritability of these mutations in seed progeny, we conducted a genotypic analysis on their progeny plants (Figure 4). We selected the T_0_ heterozygous mutants to analyze the segregation of mutations in their seed progeny including *rsz21-3*, *sc25-2*, *scl25-2*, *scl28-1*, *scl57-2*, and *rs2z39-1*. The genotyping of the T_1_ generation identified homozygous monoallelic lines for these mutants. Our genotyping analysis data reveal that the single mutants are heritable in seed progeny and we can recover homozygous mutants. These data indicate that our collection of mutants of the SR gene family will be useful for the community to use these mutants to understand the molecular underpinnings of the SR regulation in response to stress and growth cues.

## 4. Discussion

Our study provides excellent resource materials to further analyze the biological function of a family of splicing regulators in rice growth, development, and stress responses. Understanding the role of SR proteins in AS in response to environmental stresses will have implications in crop improvement [10,12,40,41]. Pre-mRNA splicing is an important regulatory mechanism during which the noncoding sequences from eukaryotic genes are excised with precision. SR proteins are RNA-binding proteins that assist the spliceosome mainly in splice site selection and are an important component of constitutive and alternative splicing. Most of our knowledge of SR proteins came from animal systems, where, in addition to mRNA splicing, these proteins are involved in mRNA export, stability, and translation; RNA metabolism; gene regulation; subcellular localization of transcripts; genome stability; chromatin binding; and miRNA processing. However, whether plant SR proteins are involved in these processes is still unknown and needs further investigation. Since plants have more SR proteins that are structurally different from mammalian SR proteins further points to some additional roles of these proteins in the plant life cycle. In plants, only Arabidopsis SR proteins have been extensively studied, in which most SR mutants are hypersensitive to abiotic stresses like salt, ABA, drought, and heavy metals [19,21,22,42]. In rice, only a couple of studies have been reported, in which overexpression of *RSZ36* and *SRp33b* changed the splicing patterns of *RSZ36* and *SRp32*, respectively [43]. In another study, Zheng et al. showed that AS plays a critical role in mineral homeostasis and SR proteins are important regulators of the Zn, Mn, and P nutrition uptake and remobilization [23]. The SR pre-mRNAs themselves are alternatively spliced under different environmental stresses and may affect the downstream AS targets [42]. This high number of SR genes in rice probably enables them to adapt to adverse environmental conditions.

**Figure 5:**
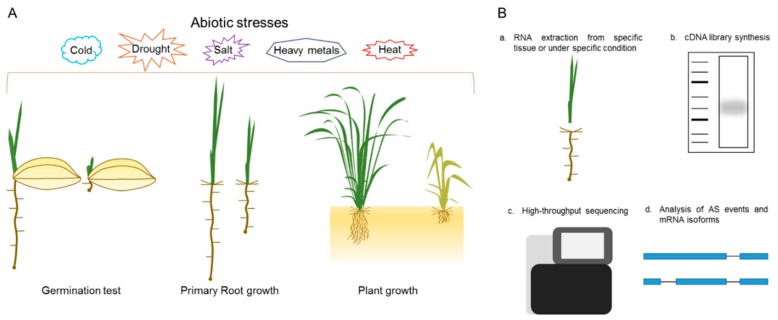
Platform for the analysis of SR mutants. (**A**) Molecular and physiological phenotyping can be done at different growth stages of SR mutants. This can be achieved by providing different abiotic stresses like salt, drought, and extreme temperatures, etc; (**B**) a general pipeline for the global analysis of alternative splicing (AS) in *SR* mutants.

Here, we proposed a platform for physiological and molecular analysis of our SR family mutants (Figure 5). For phenotypic analysis, the mutants’ response to different abiotic stresses, such as drought, salt, extreme temperatures, and heavy metals, can be investigated. The stress treatments can be conducted at various growth stages, such as seed germination, primary root inhibition, or plant growth, in soil or in hydroponic conditions (Figure 5A). These stress treatments will be applied to single or multiplex *SR* family mutants and will help to determine their molecular role during plant growth, development and in response to stress cues. The multiplex mutants were not homozygous for all targeted loci and produced combinations of single, double and triple mutants. We hypothesized that these are a good starting material for comprehensive genetic and phenotypic analysis and their segregating progeny will serve as a useful resource for the plant splicing and alternative research community. For molecular analysis, RNA-seq may be the best approach. RNA can be extracted from specific tissues (roots, shoots, and reproductive organs) or under specific conditions and used for the cDNA library synthesis. The library will be used for high-throughput sequencing and, after read alignment and splice junction predictions, can help to determine the mRNA isoforms in specific tissues or under specific conditions (Figure 5B). This approach is useful for the determination of general and specific mRNA targets of each SR family member.

## Supplementary Materials

The following are available online at https://www.mdpi.com/2073-4425/10/8/596/s1, Table S1: Synthetic fragments used as sgRNAs for targeting single and multiple SR loci, Table S2: List of oligos used for genotyping, Table S3: Summary for genotyping of SR mutants.
