# Supplementary material for: Multiplex CRISPR Mutagenesis of the Serine/Arginine-Rich (SR) Gene Family in Rice
Source: Genes (Basel). 2019 Aug 7;10(8):596. doi: 10.3390/genes10080596 (PMC6723545; doi:10.3390/genes10080596)
Supplement: Supplementary file 1 [file genes-10-00596-s001.pdf]

Supplementary material

Multiplex CRISPR Mutagenesis of the Serine/Arginine-Rich (SR) Gene Family in Rice

Haroon Butt [1], Agnieszka Piatek [1], Lixin Li [1], Anireddy S.N. Reddy [2] and Magdy M. Mahfouz [1,*]

[1] Laboratory for Genome Engineering and Synthetic Biology, Division of Biological Sciences, King Abdullah University of Science and Technology, Thuwal 23955-6900, Saudi Arabia

[2] Program in Molecular Plant Biology, Program in Cell and Molecular Biology, Department of Biology, Colorado State University, Fort Collins, CO 80523-1062, USA

* Correspondence: magdy.mahfouz@kaust.edu.sa

Keywords: splicing; alternative splicing; SR proteins; genome engineering; multiplex targeting; CRISPR/Cas9

Table S1. Synthetic fragments used as sgRNAs for targeting single and multiple SR loci.

Target Loci	5'-Sequence-3'
SR32	AACAAAGCACCAGTGGTCTAGTGGTAGAATAGTACCCTGCCACGGTACAGACCCGGGTTCGATTCCCGGCTGGTGCAAAGATCCCTCCGAGGCCACCGTTTTAGAGCTAGAAATAGCAAGTTAAAATAAGGCTAGTCCGTTATCAACTTGAAAAAGTGGCACCGAGTCGGTGCTTTTTTTTTT
SR33a	AACAAAGCACCAGTGGTCTAGTGGTAGAATAGTACCCTGCCACGGTACAGACCCGGGTTCGATTCCCGGCTGGTGCAAAAATTCCTCCAAGGCCTCCGTTTTAGAGCTAGAAATAGCAAGTTAAAATAAGGCTAGTCCGTTATCAACTTGAAAAAGTGGCACCGAGTCGGTGCTTTTTTTTTT
SR33	AACAAAGCACCAGTGGTCTAGTGGTAGAATAGTACCCTGCCACGGTACAGACCCGGGTTCGATTCCCGGCTGGTGCAGGAACCTCCCGGGCGACATCGTTTTAGAGCTAGAAATAGCAAGTTAAAATAAGGCTAGTCCGTTATCAACTTGAAAAAGTGGCACCGAGTCGGTGCTTTTTTTTTT
SR40	AACAAAGCACCAGTGGTCTAGTGGTAGAATAGTACCCTGCCACGGTACAGACCCGGGTTCGATTCCCGGCTGGTGCACATAAGGATATAGATCTTGCGTTTTAGAGCTAGAAATAGCAAGTTAAAATAAGGCTAGTCCGTTATCAACTTGAAAAAGTGGCACCGAGTCGGTGCTTTTTTTTTT
SR32/SR33a/SR33/SR40	AACAAAGCACCAGTGGTCTAGTGGTAGAATAGTACCCTGCCACGGTACAGACCCGGGTTCGATTCCCGGCTGGTGCAAAGATCCCTCCGAGGCCACCGTTTTAGAGCTAGAAATAGCAAGTTAAAATAAGGCTAGTCCGTTATCAACTTGAAAAAGTGGCACCGAGTCGGTGCAACAAAGCACCAGTGGTCTAGTGGTAGAATAGTACCCTGCCACGGTACAGACCCGGGTTCGATTCCCGGCTGGTGCAAAAATTCCTCCAAGGCCTCCGTTTTAGAGCTAGAAATAGCAAGTTAAAATAAGGCTAGTCCGTTATCAACTTGAAAAAGTGGCACCGAGTCGGTGCAACAAAGCACCAGTGGTCTAGTGGTAGAATAGTACCCTGCCACGGTACAGACCCGGGTTCGATTCCCGGCTGGTGCAGGAACCTCCCGGGCGACATCGTTTTAGAGCTAGAAATAGCAAGTTAAAATAAGGCTAGTCCGTTATCAACTTGAAAAAGTGGCACCGAGTCGGTGCAACAAAGCACCAGTGGTCTAGTGGTAGAATAGTACCCTGCCACGGTACAGACCCGGGTTCGATTCCCGGCTGGTGCACATAAGGATATAGATCTTGCGTTTTAGAGCTAGAAATAGCAAGTTAAAATAAGGCTAGTCCGTTATCAACTTGAAAAAGTGGCACCGAGTCGGTGCTTTTTTTTTT
RSZ21a	AACAAAGCACCAGTGGTCTAGTGGTAGAATAGTACCCTGCCACGGTACAGACCCGGGTTCGATTCCCGGCTGGTGCACGTGACGGCGCGGGAGCTCGGTTTTAGAGCTAGAAATAGCAAGTTAAAATAAGGCTAGTCCGTTATCAACTTGAAAAAGTGGCACCGAGTCGGTGCTTTTTTTTTT
RSZ21	AACAAAGCACCAGTGGTCTAGTGGTAGAATAGTACCCTGCCACGGTACAGACCCGGGTTCGATTCCCGGCTGGTGCAGGATCCCCGAGTGACTTCCGGTTTTAGAGCTAGAAATAGCAAGTTAAAATAAGGCTAGTCCGTTATCAACTTGAAAAAGTGGCACCGAGTCGGTGCTTTTTTTTTT
RSZ23	AACAAAGCACCAGTGGTCTAGTGGTAGAATAGTACCCTGCCACGGTACAGACCCGGGTTCGATTCCCGGCTGGTGCAGAACCTGGATCCGCGCGTGAGTTTTAGAGCTAGAAATAGCAAGTTAAAATAAGGCTAGTCCGTTATCAACTTGAAAAAGTGGCACCGAGTCGGTGCTTTTTTTTTT
RSZ21a/RSZ21/RSZ23	AACAAAGCACCAGTGGTCTAGTGGTAGAATAGTACCCTGCCACGGTACAGACCCGGGTTCGATTCCCGGCTGGTGCACGTGACGGCGCGGGAGCTCGGTTTTAGAGCTAGAAATAGCAAGTTAAAATAAGGCTAGTCCGTTATCAACTTGAAAAAGTGGCACCGAGTCGGTGCAACAAAGCACCAGTGGTCTAGTGGTAGAATAGTACCCTGCCACGGTACAGACCCGGGTTCGATTCCCGGCTGGTGCAGGATCCCCGAGTGACTTCCGGTTTTAGAGCTAGAAATAGCAAGTTAAAATAAGGCTAGTCCGTTATCAACTTGAAAAAGTGGCACCGAGTCGGTGCAACAAAGCACCAGTGGTCTAGTGGTAGAATAGTACCCTGCCACGGTACAGACCCGGGTTCGATTCCCGGCTGGTGCAGAACCTGGATCCGCGCGTGAGTTTTAGAGCTAGAAATAGCAAGTTAAAATAAGGCTAGTCCGTTATCAACTTGAAAAAGTGGCACCGAGTCGGTGCTTTTTTTTTT
SC25	AACAAAGCACCAGTGGTCTAGTGGTAGAATAGTACCCTGCCACGGTACAGACCCGGGTTCGATTCCCGGCTGGTGCACCTCGGCCGCTACGGCCCACGTTTTAGAGCTAGAAATAGCAAGTTAAAATAAGGCTAGTCCGTTATCAACTTGAAAAAGTGGCACCGAGTCGGTGCTTTTTTTTTT
SC32	AACAAAGCACCAGTGGTCTAGTGGTAGAATAGTACCCTGCCACGGTACAGACCCGGGTTCGATTCCCGGCTGGTGCAATGTCGCGCTTCGGCCGCTCGTTTTAGAGCTAGAAATAGCAAGTTAAAATAAGGCTAGTCCGTTATCAACTTGAAAAAGTGGCACCGAGTCGGTGCTTTTTTTTTT
SC34	AACAAAGCACCAGTGGTCTAGTGGTAGAATAGTACCCTGCCACGGTACAGACCCGGGTTCGATTCCCGGCTGGTGCACTTCGGGAGGTCGGGGCCGCGTTTTAGAGCTAGAAATAGCAAGTTAAAATAAGGCTAGTCCGTTATCAACTTGAAAAAGTGGCACCGAGTCGGTGCTTTTTTTTTT
SC25/SC32/SC34	AACAAAGCACCAGTGGTCTAGTGGTAGAATAGTACCCTGCCACGGTACAGACCCGGGTTCGATTCCCGGCTGGTGCACCTCGGCCGCTACGGCCCACGTTTTAGAGCTAGAAATAGCAAGTTAAAATAAGGCTAGTCCGTTATCAACTTGAAAAAGTGGCACCGAGTCGGTGCAACAAAGCACCAGTGGTCTAGTGGTAGAATAGTACCCTGCCACGGTACAGACCCGGGTTCGATTCCCGGCTGGTGCAATGTCGCGCTTCGGCCGCTCGTTTTAGAGCTAGAAATAGCAAGTTAAAATAAGGCTAGTCCGTTATCAACTTGAAAAAGTGGCACCGAGTCGGTGCAACAAAGCACCAGTGGTCTAGTGGTAGAATAGTACCCTGCCACGGTACAGACCCGGGTTCGATTCCCGGCTGGTGCACTTCGGGAGGTCGGGGCCGCGTTTTAGAGCTAGAAATAGCAAGTTAAAATAAGGCTAGTCCGTTATCAACTTGAAAAAGTGGCACCGAGTCGGTGCTTTTTTTTTT
SCL25	AACAAAGCACCAGTGGTCTAGTGGTAGAATAGTACCCTGCCACGGTACAGACCCGGGTTCGATTCCCGGCTGGTGCAGAGGACCTTCGTCGGCCATTGTTTTAGAGCTAGAAATAGCAAGTTAAAATAAGGCTAGTCCGTTATCAACTTGAAAAAGTGGCACCGAGTCGGTGCTTTTTTTTTT
SCL26	AACAAAGCACCAGTGGTCTAGTGGTAGAATAGTACCCTGCCACGGTACAGACCCGGGTTCGATTCCCGGCTGGTGCAAGTCCTCGTGGGCGCTATGGGTTTTAGAGCTAGAAATAGCAAGTTAAAATAAGGCTAGTCCGTTATCAACTTGAAAAAGTGGCACCGAGTCGGTGCTTTTTTTTTT
SCL28	AACAAAGCACCAGTGGTCTAGTGGTAGAATAGTACCCTGCCACGGTACAGACCCGGGTTCGATTCCCGGCTGGTGCACCGGGTACCGGAGCCGGAGCGTTTTAGAGCTAGAAATAGCAAGTTAAAATAAGGCTAGTCCGTTATCAACTTGAAAAAGTGGCACCGAGTCGGTGCTTTTTTTTTT
SCL25/SCL26/SCL28	AACAAAGCACCAGTGGTCTAGTGGTAGAATAGTACCCTGCCACGGTACAGACCCGGGTTCGATTCCCGGCTGGTGCAGAGGACCTTCGTCGGCCATTGTTTTAGAGCTAGAAATAGCAAGTTAAAATAAGGCTAGTCCGTTATCAACTTGAAAAAGTGGCACCGAGTCGGTGCAACAAAGCACCAGTGGTCTAGTGGTAGAATAGTACCCTGCCACGGTACAGACCCGGGTTCGATTCCCGGCTGGTGCAAGTCCTCGTGGGCGCTATGGGTTTTAGAGCTAGAAATAGCAAGTTAAAATAAGGCTAGTCCGTTATCAACTTGAAAAAGTGGCACCGAGTCGGTGCAACAAAGCACCAGTGGTCTAGTGGTAGAATAGTACCCTGCCACGGTACAGACCCGGGTTCGATTCCCGGCTGGTGCACCGGGTACCGGAGCCGGAGCGTTTTAGAGCTAGAAATAGCAAGTTAAAATAAGGCTAGTCCGTTATCAACTTGAAAAAGTGGCACCGAGTCGGTGCTTTTTTTTTT

Target Locus	5'-Sequence-3'
SCL30a/SCL30	AACAAAGCACCAGTGGTCTAGTGGTAGAATAGTACCCTGCCACGGTACAGACCCGGGTTCGATTCCCGGCTGGTGCAAGTCCCCCTAGGAGGGGATAGTTTTAGAGCTAGAAATAGCAAGTTAAAATAAGGCTAGTCCGTTATCAACTTGAAAAAGTGGCACCGAGTCGGTGCTTTTTTTTTT
SCL57	AACAAAGCACCAGTGGTCTAGTGGTAGAATAGTACCCTGCCACGGTACAGACCCGGGTTCGATTCCCGGCTGGTGCAGGAGCCGCAGCCCCAGCAAGGTTTTAGAGCTAGAAATAGCAAGTTAAAATAAGGCTAGTCCGTTATCAACTTGAAAAAGTGGCACCGAGTCGGTGCTTTTTTTTTT
SCL30a/SCL30/SCL57	AACAAAGCACCAGTGGTCTAGTGGTAGAATAGTACCCTGCCACGGTACAGACCCGGGTTCGATTCCCGGCTGGTGCAAGTCCCCCTAGGAGGGGATAGTTTTAGAGCTAGAAATAGCAAGTTAAAATAAGGCTAGTCCGTTATCAACTTGAAAAAGTGGCACCGAGTCGGTGCAACAAAGCACCAGTGGTCTAGTGGTAGAATAGTACCCTGCCACGGTACAGACCCGGGTTCGATTCCCGGCTGGTGCAGGAGCCGCAGCCCCAGCAAGGTTTTAGAGCTAGAAATAGCAAGTTAAAATAAGGCTAGTCCGTTATCAACTTGAAAAAGTGGCACCGAGTCGGTGCTTTTTTTTTT
RS2Z36/RS2Z38	AACAAAGCACCAGTGGTCTAGTGGTAGAATAGTACCCTGCCACGGTACAGACCCGGGTTCGATTCCCGGCTGGTGCATGTTTTAACTGTGGGATTGAGTTTTAGAGCTAGAAATAGCAAGTTAAAATAAGGCTAGTCCGTTATCAACTTGAAAAAGTGGCACCGAGTCGGTGCTTTTTTTTTT
RS2Z37	AACAAAGCACCAGTGGTCTAGTGGTAGAATAGTACCCTGCCACGGTACAGACCCGGGTTCGATTCCCGGCTGGTGCATTCCTCGCGCACCCGGACCCGTTTTAGAGCTAGAAATAGCAAGTTAAAATAAGGCTAGTCCGTTATCAACTTGAAAAAGTGGCACCGAGTCGGTGCTTTTTTTTTT
RS2Z39	AACAAAGCACCAGTGGTCTAGTGGTAGAATAGTACCCTGCCACGGTACAGACCCGGGTTCGATTCCCGGCTGGTGCACCCGGGATGCAAATGATGCGGTTTTAGAGCTAGAAATAGCAAGTTAAAATAAGGCTAGTCCGTTATCAACTTGAAAAAGTGGCACCGAGTCGGTGCTTTTTTTTTT
RS2Z36/RS2Z37/RS2Z38/RS2Z39	AACAAAGCACCAGTGGTCTAGTGGTAGAATAGTACCCTGCCACGGTACAGACCCGGGTTCGATTCCCGGCTGGTGCATGTTTTAACTGTGGGATTGAGTTTTAGAGCTAGAAATAGCAAGTTAAAATAAGGCTAGTCCGTTATCAACTTGAAAAAGTGGCACCGAGTCGGTGCAACAAAGCACCAGTGGTCTAGTGGTAGAATAGTACCCTGCCACGGTACAGACCCGGGTTCGATTCCCGGCTGGTGCATTCCTCGCGCACCCGGACCCGTTTTAGAGCTAGAAATAGCAAGTTAAAATAAGGCTAGTCCGTTATCAACTTGAAAAAGTGGCACCGAGTCGGTGCAACAAAGCACCAGTGGTCTAGTGGTAGAATAGTACCCTGCCACGGTACAGACCCGGGTTCGATTCCCGGCTGGTGCACCCGGGATGCAAATGATGCGGTTTTAGAGCTAGAAATAGCAAGTTAAAATAAGGCTAGTCCGTTATCAACTTGAAAAAGTGGCACCGAGTCGGTGCTTTTTTTTTT
RS29	AACAAAGCACCAGTGGTCTAGTGGTAGAATAGTACCCTGCCACGGTACAGACCCGGGTTCGATTCCCGGCTGGTGCAGCTATCCTTTTGGCCCTGGGGTTTTAGAGCTAGAAATAGCAAGTTAAAATAAGGCTAGTCCGTTATCAACTTGAAAAAGTGGCACCGAGTCGGTGCTTTTTTTTTT
RS33	AACAAAGCACCAGTGGTCTAGTGGTAGAATAGTACCCTGCCACGGTACAGACCCGGGTTCGATTCCCGGCTGGTGCAGAGCGCCTCTTCAGCAAATAGTTTTAGAGCTAGAAATAGCAAGTTAAAATAAGGCTAGTCCGTTATCAACTTGAAAAAGTGGCACCGAGTCGGTGCTTTTTTTTTT
RS29/RS33	AACAAAGCACCAGTGGTCTAGTGGTAGAATAGTACCCTGCCACGGTACAGACCCGGGTTCGATTCCCGGCTGGTGCAGCTATCCTTTTGGCCCTGGGGTTTTAGAGCTAGAAATAGCAAGTTAAAATAAGGCTAGTCCGTTATCAACTTGAAAAAGTGGCACCGAGTCGGTGCAACAAAGCACCAGTGGTCTAGTGGTAGAATAGTACCCTGCCACGGTACAGACCCGGGTTCGATTCCCGGCTGGTGCAGAGCGCCTCTTCAGCAAATAGTTTTAGAGCTAGAAATAGCAAGTTAAAATAAGGCTAGTCCGTTATCAACTTGAAAAAGTGGCACCGAGTCGGTGCTTTTTTTTTT
U3 promoter	GACCATGATTACGCCAAGCTTAAGGAATCTTTAAACATACGAACAGATCACTTAAAGTTCTTCTGAAGCAACTTAAAGTTATCAGGCATGCATGGATCTTGGAGGAATCAGATGTGCAGTCAGGGACCATAGCACAAGACAGGCGTCTTCTACTGGTGCTACCAGCAAATGCTGGAAGCCGGGAACACTGGGTACGTTGGAAACCACGTGATGTGAAGAAGTAAGATAAACTGTAGGAGAAAAGCATTTCGTAGTGGGCCATGAAGCCTTTCAGGACATGTATTGCAGTATGGGCCGGCCCATTACGCAATTGGACGACAACAAAGACTAGTATTAGTACCACCTCGGCTATCCACATAGATCAAAGCTGATTTAAAAGAGTTGTGCAGATGATCCGTGGCA

pre-tRNA: yellow backgroundgRNA spacer: gray backgroundScaffold RNA: green backgroundpolIII terminator: red background

Table S2: List of oligos used for genotyping

Oligo Name	5'-Sequence-3'
SR32_F	GGGAAATAAGTAAGGCATGGC
SR32_R	GTAACCAGAACTGTGTGAAGTG
SR33a_F	CACTTGGAGAGATTCCACCAG
SR33a_R	CACCTCGCTACCTCCTATTC
SR33_F	GTGGTCCAGTTTGGGGTTCAG
SR33_R	GTAGCTGCTAGAAGCATACAG
SR40_F	GTACTAAAGGATGGACCACTTG
SR40_R	GAATAGTTAGGTACCATGCG
RSZ21a_F	CTCTCGCCTCCCCCCTCTTAC
RSZ21a_R	GCCTGGATGCCATAGCTGGG
RSZ21_F	CAGCTTCAGATTTCTGACACC
RSZ21_R	CTTATAGTACAAGGGGTACC
RSZ23_F	GTACTCTCCAGATCCCCCGC
RSZ23_R	CCCAATCCCACCAGTAGAAC
SC25_F	GCCGCGTCATCTCTTCTCTTC
SC25_R	GAACGCGAACCCTCTCGAATC
SC32_F	GTGGCCACCCTCACCCTAC
SC32_R	CAGCCGACCTAATCAGCTCCC
SC34_F	CTCCACTGCACACACTCGCTG
SC34_R	GAATCCCCTCGAGTCCCCAGTC
SCL25_F	CTTAAGCCGGTTATAGCGGCC
SCL25_R	CCCTTCTCTTCAACAGACTTG
SCL26_F	GCTTGTTTTATTCCCTACCAG
SCL26_R	CCTTACAGTTACGAACTTCTG
SCL28_F	ACCTCTCCTCGCCCAAGCC
SCL28_R	GTAAGCCCTAGATCAAATGGGC
SCL30a_F	GTACTTGAAGTTACCTATGGG
SCL30a_R	GGAACTCTAAGATCCTCCGC
SCL30_F	GTTGGCCCAACATATTTTCTG
SCL30_R	CTGCATAACATCAGAACAGC
SCL57_F	CTGCCTAGCAAGTAGAATACC
SCL57_R	CCAGACATGACAGAAGTTAGAC
RS2Z36_F	CCTTGATGGCAGGGATGTTG
RS2Z36_R	GACCGGCTGATATAAAGAATG
RS2Z37_F	CCCTAGCTTCTCTTGTAGATC
RS2Z37_R	CAGCATGCTGCTGGCCTAGAC
RS2Z38_F	GGGATGTTGATGGAAGCCGC
RS2Z38_R	CTGGACCTAGATCGGCTGATAG
RS2Z39_F	CACAGACTAGTGGATGTTCCC
RS2Z39_R	CCCTCTCTCCACAGCCATAG
RS29_F	GCAGGTGCAGTACTCTTCTG
RS29_R	GTGGCCTCTTCCTGTGTTTC
RS33_F	CCTAGCATTGGCTATGGTGG
RS33_R	CAAATGGAGTACCCACTCGC

Table S3: Summary for genotyping of SR mutants

Target gene(s)	No. of plants genotyped	No. of plants mutated
SR32	20	1
SR33a	5	3
SR33	5	4
SR40	5	3
SR32/SR33a/SR33/SR40	10	None of the plant is homozygous mutant for all loci
RSZ21a	5	1
RSZ21	5	4
RSZ23	5	2
RSZ21a/RSZ21/RSZ23	10	None of the plant is homozygous mutant for all loci
SC25	5	4
SC32	5	5
SC34	5	3
SC25/SC32/SC34	10	None of the plant is homozygous mutant for all loci
SCL25	8	4
SCL26	5	4
SCL28	4	1
SCL25/SCL26/SCL28	10	None of the plant is homozygous mutant for all loci
SCL30a/SCL30	5	None of the plant is homozygous mutant for all loci
SCL57	5	5
SCL30a/SCL30/SCL57	5	None of the plant is homozygous mutant for all loci
RS2Z36/RS2Z38	5	1 double mutant
RS2Z37	5	5
RS2Z39	5	5
RS2Z36/RS2Z37/RS2Z38/RS2Z39	10	None of the plant is homozygous mutant for all loci
RS29	5	2
RS33	5	3
RS29/RS33	10	1 double mutant
